# Supplementary material for: A 28 Day Clinical Assessment of a Lactic Acid-containing Antimicrobial Intimate Gel Wash Formulation on Skin Tolerance and Impact on the Vulvar Microbiome
Source: Antibiotics (Basel). 2020 Feb 1;9(2):55. doi: 10.3390/antibiotics9020055 (PMC7168340; doi:10.3390/antibiotics9020055)
Supplement: Supplementary file 1 [file antibiotics-09-00055-s001.pdf]

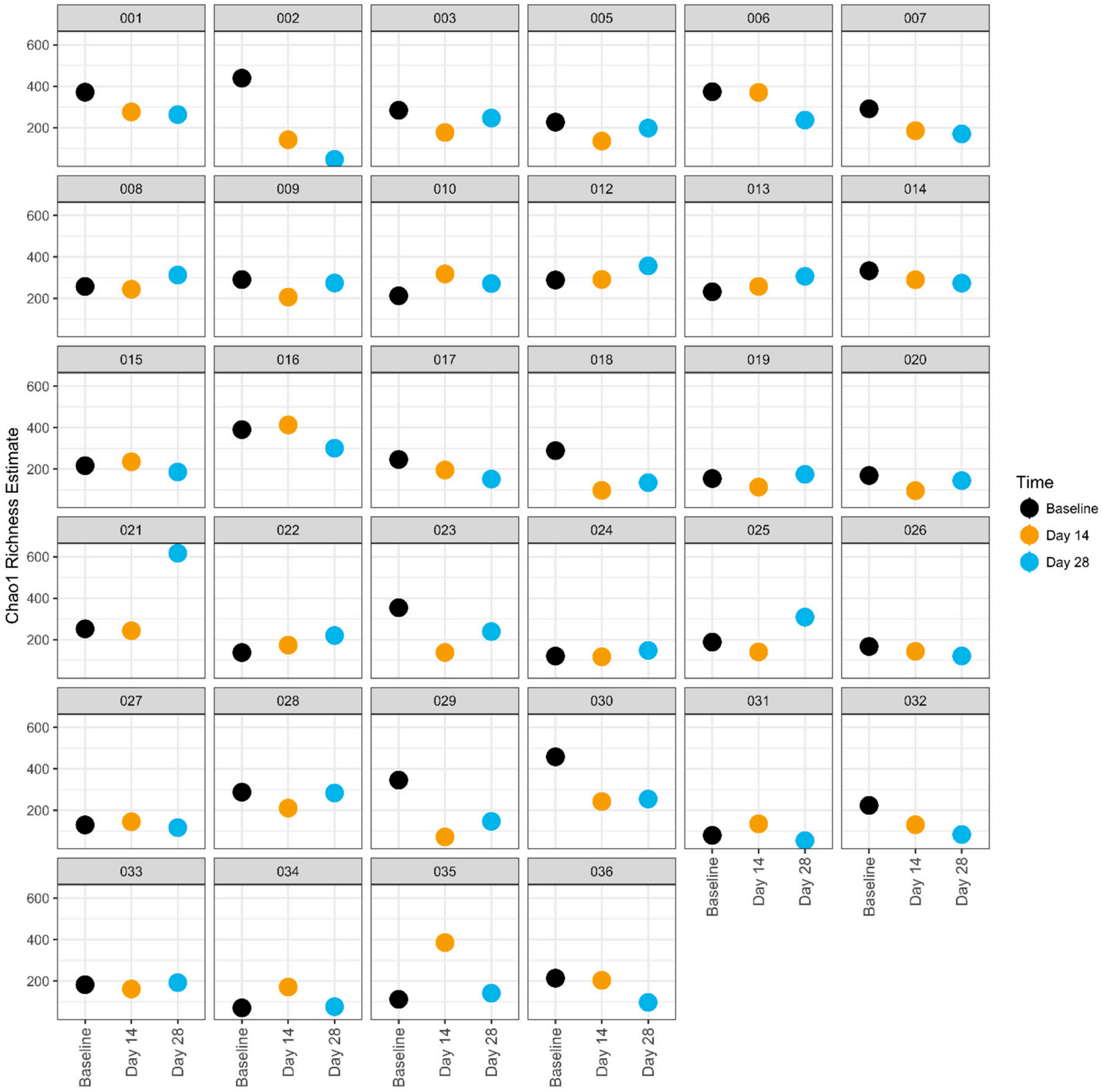

Figure S1: Individual Subject Bacteria Chao1 Richness Plot

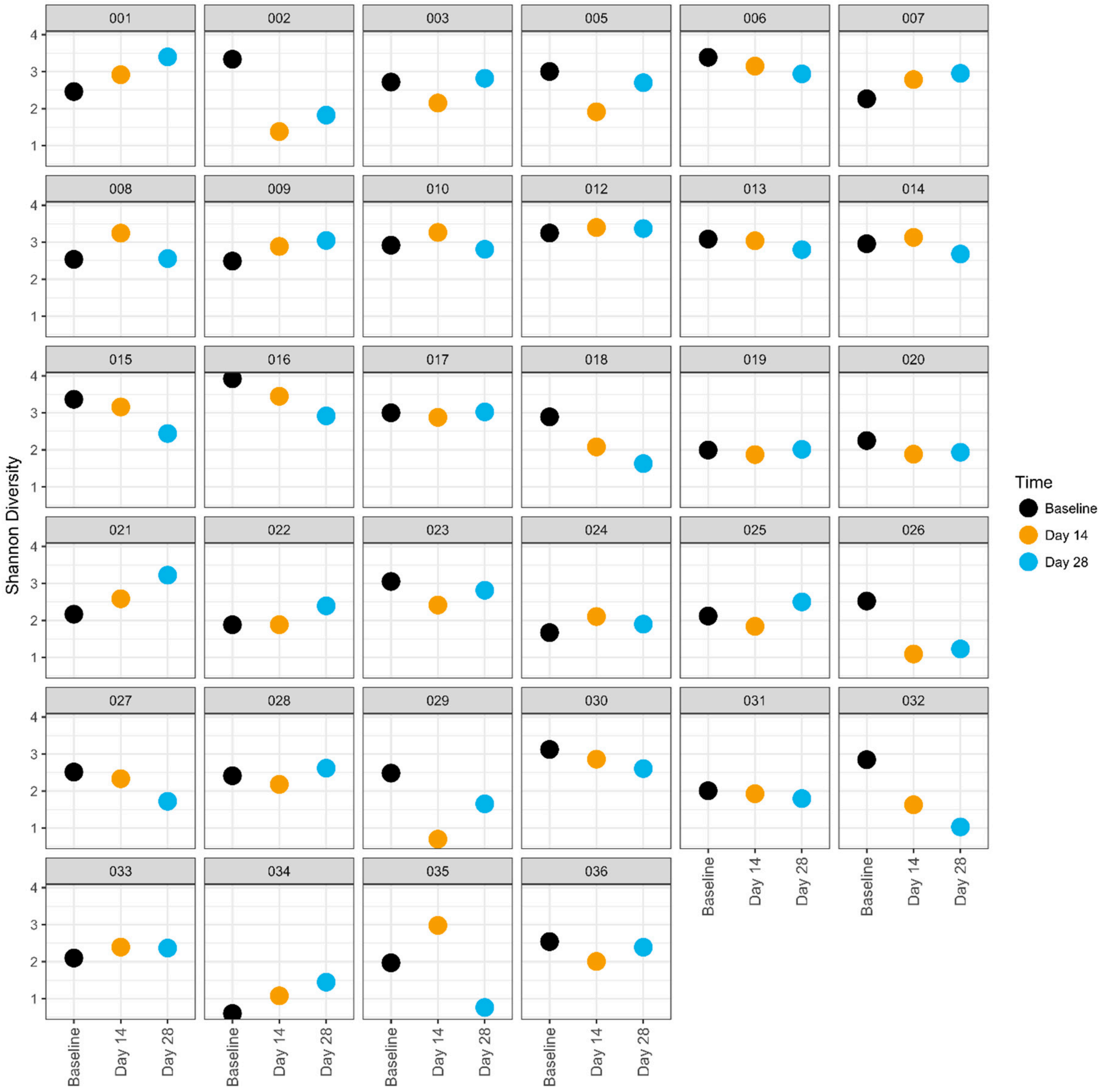

Figure S2: Individual Subject Bacteria Shannon Diversity Plot

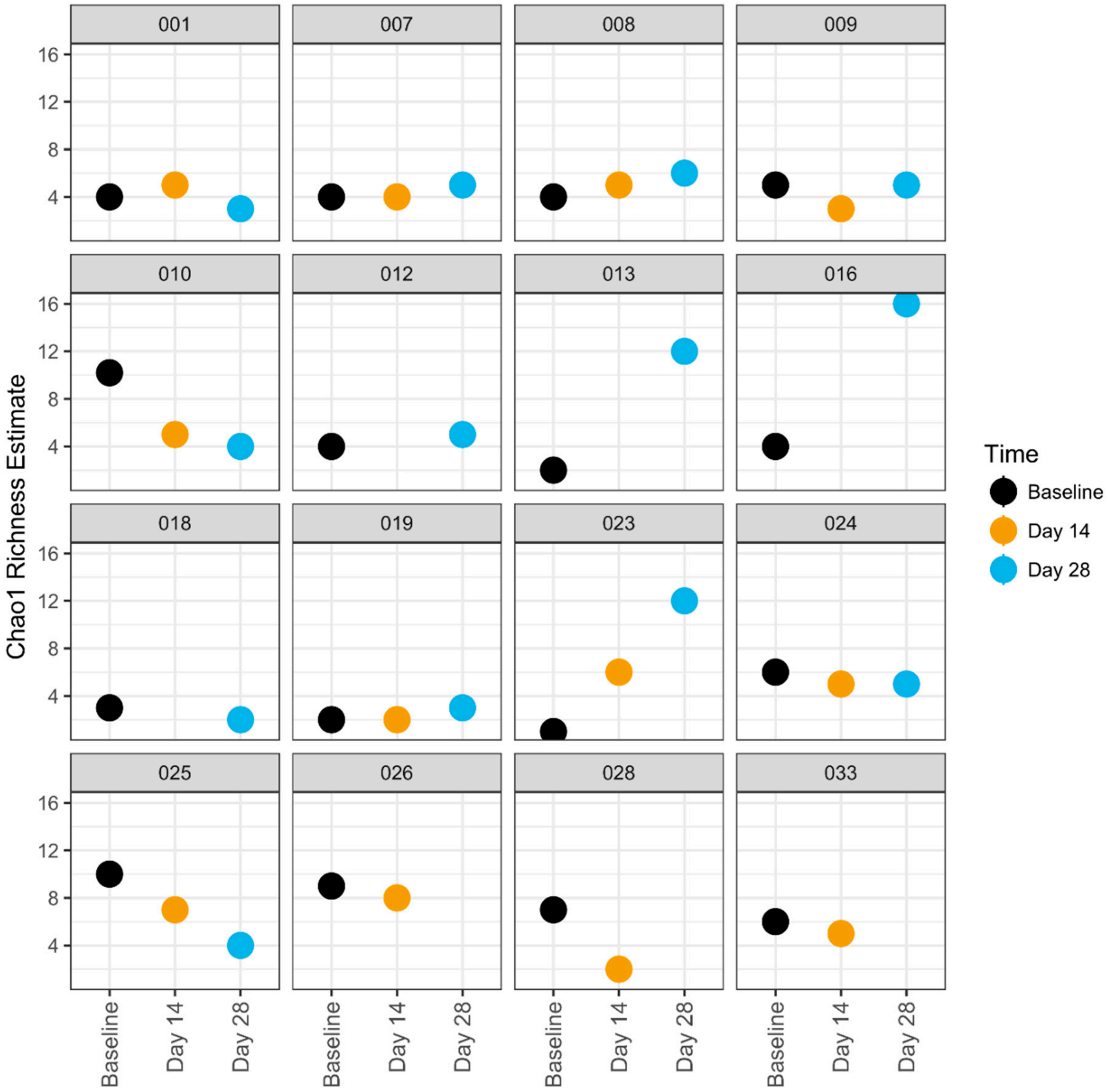

Figure S3: Individual Subject Fungal Chao1 Richness Plot

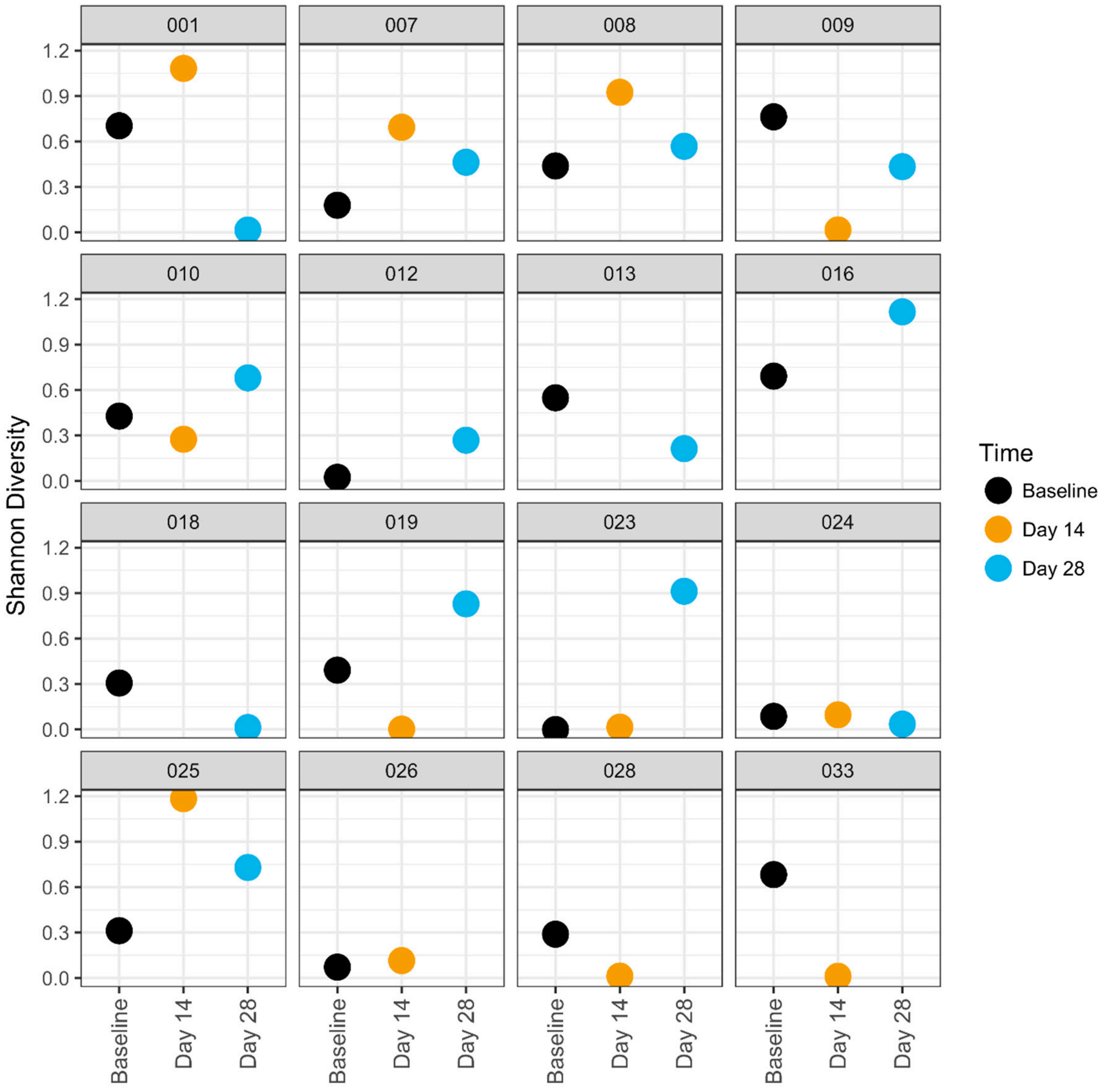

Figure S4: Individual Subject Fungal Shannon Diversity Plot
